# Methane Found in Well Water Near Fracking Sites

**DOI:** 10.1289/ehp.119-a289

**Published:** 2011-07-01

**Authors:** David C. Holzman

**Affiliations:** David C. Holzman writes on science, medicine, energy, economics, and cars from Lexington and Wellfleet, MA. His work has appeared in *Smithsonian*, *The Atlantic Monthly*, and the *Journal of the National Cancer Institute*.

In a study of 68 private drinking water wells in northeastern Pennsylvania and New York, methane contamination rose sharply with proximity to natural gas drilling and hydraulic fracturing (“fracking”) sites.^1^ The average methane concentration in shallow groundwater in active drilling areas fell within the defined action level (> 10 mg/L but < 28 mg/L) for hazard mitigation recommended by the U.S. Department of the Interior, and the maximum (64 mg/L) was well beyond that threshold, according to the report. However, the researchers found no evidence of fracturing fluids. Principal investigator Robert B. Jackson of Duke University says fracking has been conducted in the sampled region since about 2008. The team sampled the water supplies in 2010.

The researchers measured concentrations of gases and certain isotopes of carbon in methane and other hydrocarbons to distinguish the ancient thermogenic gas stores sought in drilling operations from methane generated by microbial degradation of organic matter. The closer the well was to an active drilling site, the more likely it was the methane detected was thermogenic.

Flammable levels of natural gas are common in water supplies, and explosions—even reports of flammable drinking water—have occurred near fracking sites, says Abrahm Lustgarten, a reporter for Propublica who has investigated gas drilling across the United States. But no peer-reviewed studies have investigated health effects of chronic ingestion of small amounts of methane, Jackson says.^2^

John Hanger, a former head of the Pennsylvania Department of Environmental Protection (DEP), blames poor gas well construction or design, not fracking, for methane contamination noted to date. He says repairs or plugging of gas wells eliminated contamination in 14 of 19 previously contaminated water wells tested in 2010 by the DEP. But Jackson maintains fracking cannot be ruled out as a cause, given the high pressures used in the practice.

Just how likely are leaks? Based on a non-peer-reviewed survey of the five states that systematically report incidents at wells where fracking occurs and where complaints have spurred inspections, Ronald E. Bishop, a lecturer in chemistry and biochemistry at the State University of New York, Oneonta, estimates nearly 2% of such gas wells may end up contaminating groundwater with fracking fluids.^3^ Bishop says 50% of new natural gas wells recently inspected in Quebec leaked methane.^4^

Hanger says Pennsylvania has enacted strict standards for design, construction, and materials used in gas wells, which became effective in February 2011.^5^ The state also requires testing, monitoring, and disclosure of chemicals used in fracking. But Amy Mall, a senior policy analyst with the Natural Resources Defense Council, says state fracking regulations vary widely, and a federal law passed in 2005 exempts fracking from the Safe Drinking Water Act and companies from disclosing chemicals used during the process.

## References

[1] Osborn SG Methane contamination of drinking water accompanying gas-well drilling and hydraulic fracturing. Proc Natl Acad Sci USA 108 20 8172 8176 2011 doi:10.1073/pnas.1100682108 21555547 PMC3100993

[2] Jackson RB, et al. Research and Policy Recommendations for Hydraulic Fracturing and Shale-Gas Extraction. Durham, NC:Center on Global Change, Duke University (2011). Available: http://tinyurl.com/6kep5fp [accessed 17 Jun 2011].

[3] Bishop RE. Chemical and Biological Risk Assessment for Natural Gas Extraction in New York. Oneonta, NY:State University of New York College at Oneonta; Cooperstown, NY:Sustainable Otsego (28 Mar 2011). Available: http://tinyurl.com/5wp4ybg [accessed 17 Jun 2011].

[4] Re: Réponses aux questions DQ28 (1-5) [in French]. Letter from Jean-Yves Laliberté to Monique Gélinas. 7 December 2010. Available: http://tinyurl.com/5tun62v [accessed 17 Jun 2011].

[5] Commonwealth of Pennsylvania. The Pennsylvania Code. Title 25: Environmental Protection, Chapter 78: Oil and Gas Wells. Mechanicsburg, PA:Fry Communications, Inc.; Madison, WI:Legislative Reference Bureau. Available: http://tinyurl.com/69qfaws [accessed 17 Jun 2011].

